# Dual Targeting with Cell Surface Electrical Charge and Folic Acid via Superparamagnetic Fe_3_O_4_@Cu_2–x_S for Photothermal Cancer Cell Killing

**DOI:** 10.3390/cancers13215275

**Published:** 2021-10-21

**Authors:** Zicheng Deng, Jou Lin, Sergey L. Bud’ko, Brent Webster, Tanya V. Kalin, Vladimir V. Kalinichenko, Donglu Shi

**Affiliations:** 1 The Materials Science and Engineering Program, College of Engineering and Applied Science, University of Cincinnati, Cincinnati, OH 45221, USA; dengzh@mail.uc.edu (Z.D.); lin2jo@mail.uc.edu (J.L.); websteba@mail.uc.edu (B.W.); 2 Center for Lung Regenerative Medicine, Cincinnati Children’s Hospital Medical Center, 3333 Burnet Avenue, Cincinnati, OH 45229, USA; 3 Division of Pulmonary Biology, Cincinnati Children’s Hospital Medical Center, 3333 Burnet Avenue, Cincinnati, OH 45229, USA; tatiana.kalin@cchmc.org; 4 Division of Materials Science and Engineering, Ames Laboratory, Ames, IA 50011, USA; budko@ameslab.gov; 5 Department of Pediatrics, College of Medicine, University of Cincinnati and Cincinnati Children’s Hospital Medical Center, 3333 Burnet Avenue, Cincinnati, OH 45229, USA

**Keywords:** superparamagnetic nanoparticles, cancer cell photothermal therapy, surface charge targeting, folic acid targeting, vitamin E TPGS modification

## Abstract

**Simple Summary:**

There are two critical issues in cancer hyperthermia: (1) photothermal effect and (2) cancer cell targeting efficiency. While the former can be addressed by rendering the nano carriers with significant IR absorptions, the latter is dealt with using a novel dual-targeting strategy. In this study, the Fe_3_O_4_ nanoparticle was coated with a shell of Cu_2–x_S; the resulting Fe_3_O_4_@Cu_2–x_S exhibited strong IR absorption for enhanced photothermal cancer cell killing. The Fe_3_O_4_@Cu_2–x_S nanoparticles are surface functionalized with amphiphilic polyethylenimine (LA-PEI) and Folic acid-TPGS (FA-TPGS) for two purposes: (1) the PEI surface coating renders the particles positively charged, enabling them to effectively bind with negatively-charged cancer cells for more intimate nano/bio contact resulting in much stronger cancer cell ablation; (2) the folic acid modification further increases the targeting efficiency via the folic receptors on the cancer cell surface. Dual-targeting with the surface electrical charge and the tumor-specific folic acid synergistically facilitates both passive and active targeting for significantly improved photothermal killing.

**Abstract:**

A major challenge in cancer therapy is to achieve high cell targeting specificity for the highest therapeutic efficacy. Two major approaches have been shown to be quite effective, namely, (1) bio-marker mediated cell targeting, and (2) electrical charge driven cell binding. The former utilizes the tumor-specific moieties on nano carrier surfaces for active targeting, while the latter relies on nanoparticles binding onto the cancer cell surfaces due to differences in electrical charge. Cancer cells are known for their hallmark metabolic pattern: high rates of glycolysis that lead to negatively charged cell surfaces. In this study, the nanoparticles of Fe_3_O_4_@Cu_2–x_S were rendered positively charged by conjugating their surfaces with different functional groups for strong electrostatic binding onto the negatively-charged cancer cells. In addition to the positively charged surfaces, the Fe_3_O_4_@Cu_2–x_S nanoparticles were also modified with folic acid (FA) for biomarker-based cell targeting. The dual-targeting approach synergistically utilizes the effectiveness of both charge- and biomarker-based cell binding for enhanced cell targeting. Further, these superparamagnetic Fe_3_O_4_@Cu_2–x_S nanoparticles exhibit much stronger IR absorptions compared to Fe_3_O_4_, therefore much more effective in photothermal therapy.

## 1. Introduction

Hyperthermia therapy has been shown to be an effective and efficient cancer treatment when applied locally to kill the cancer cells in a tumor-isolated fashion without adverse effects on healthy cells and tissues [1]. The key in a successful photothermal therapy is the targeted delivery of therapeutic agents, such as the photothermal nanoparticles, to tumors in a precision manner. Upon application of near-infrared (NIR) laser, the nanoparticles, typically gold or iron oxide, that are taken up by the tumor, can raise temperature to hyperthermic levels (~45 °C) for ablation of the targeted cancer cells [2]. In recent years, a variety of nanoparticles have been developed with multifunctionalities for medical diagnosis and therapeutics, among which the iron-oxide nanoparticles exhibit pronounced photothermal effects and, therefore, are most widely applied for photothermal therapy (PTT) [3,4,5,6]. Specifically, the superparamagnetic Fe_3_O_4_ nanoparticles have been extensively studied for biomedical applications, such as gene or drug delivery, magnetic resonance imaging (MRI), and magnetically-guided targeting [7,8,9,10,11,12]. With these unique nanoparticles, various cancer therapeutic strategies have been developed utilizing some of their fascinating properties, such as chemical stability, bio-compatibility, and strong photothermal effects. Recently, enhanced NIR absorption has been observed in modified iron-oxide nanoparticles for much stronger photothermal effects [13,14,15]. As is well-known, the Fe_3_O_4_ nanoparticles have a strong UV absorption, but it gradually decreases in the visible region without any noticeable NIR peaks [16]. 

Our previous works have shown that, by decorating the Fe_3_O_4_ nanoparticles with the Cu_2–x_S shells, the modified Fe_3_O_4_@Cu_2–x_S nanoparticles exhibit pronounced IR absorptions with broad absorption peaks near 1160 nm. This is due to the fact that the Cu_2–x_S nanocrystal has a tunable localized surface plasmon resonance (LSPR) in the IR region [16,17]. The enhanced IR absorption in Fe_3_O_4_@Cu_2–x_S can provide much greater photothermal effect in cancer hyperthermia therapy. 

To achieve maximum therapeutic effects, cell targeting is critically required for high uptake of the nanoparticles into tumor cells via systemic intravenous administration. Selective delivery of therapeutic agents into tumor lesions has been a key challenge for the successful management of cancers. To address this critical issue in cell targeting, an electrical charge-based targeting method has been developed [18]. This unique targeting method is based on the so-called Warburg effect that characterizes the cancer cells with high rate of glycolysis. Normal cells typically depend on the mitochondrial oxidative phosphorylation process to generate Adenosine triphosphate (ATP). However, all cancer cells exhibit negative surface charges that are associated with their metabolic behavior: they constantly secrete lactic acid, resulting in the cross-membrane movement of lactate, an end product of the glycolysis pathway in hypoxia. Therefore, the increase of glycolysis levels in cancer cells causes increased glucose uptake and lactate secretion levels, exceeding that of normal cells [19]. The cross-membrane movement of lactate in cancer cells also causes the loss of labile inorganic cations that form lactate salts and acids [19]. Consequently, the cancer cell surfaces are left with a net of negative electrical charges [18,20,21]. If the nanoparticles can be rendered positively charged, they are able to electrostatically bind onto the cancer cells for the detection, targeting, and effective cell killing via PTT. In our previous work, we succeeded in photothermal therapy with only charge-based cell targeting. The positively-charged Fe_3_O_4_ nanoparticles were found to bind onto cancer cells more efficiently compared to the negatively-charged counterparts. This is due to the Coulomb force attraction between the nanoparticles and tumor cells with the opposite charges [18,19,22]. Surface charge-mediated cancer cell targeting has also been utilized to engineer a nanoprobe for the detection of circulating tumor cells (CTC) in clinical blood [23,24]. 

Furthermore, active targeting has been achieved by using the tumor-specific ligands to target the cell surface molecules or receptors [25]. Folic acid (FA) is considered one of the most suitable targeting ligands for cancer therapy due to the folate receptor being overexpressed on many cancer cell types [26,27,28]. Recent research on the magnetic nanoparticles has shown that FA modification is an effective strategy to enhance cancer cell targeting efficiency [29,30,31,32,33]. 

To enhance PTT efficiency in this study, we carried out photothermal experiments with two new strategies: (1) the Fe_3_O_4_ nanoparticles were surface-modified with CuS to develop Fe_3_O_4_@Cu_2–x_S with enhanced NIR absorptions for stronger photothermal cell killing; (2) in addition to bio-targeting with FA, the nanoparticles were rendered positively charged to achieve the so-called dual cell targeting for increased cell-specific binding. It is well-known that Fe_3_O_4_ has strong UV absorption, but shows no peaks in the IR region. For more efficient photothermal heating, we have developed a core–shell structure with Cu_2–x_S forming a shell on the core of Fe_3_O_4_. In this hybrid structure, while Fe_3_O_4_ provides the superparamagnetic property for the photothermal effect, the Cu_2–x_S shell on Fe_3_O_4_ renders the system with pronounced IR absorptions for further enhancement of the PTT efficacy. These supermagnetic nanoparticles have been widely used in medical diagnostics and therapeutics, such as the magnetic resonance imaging (MRI), photodynamic therapy, and magnetic targeting [34,35,36,37]. 

**Figure 1:**
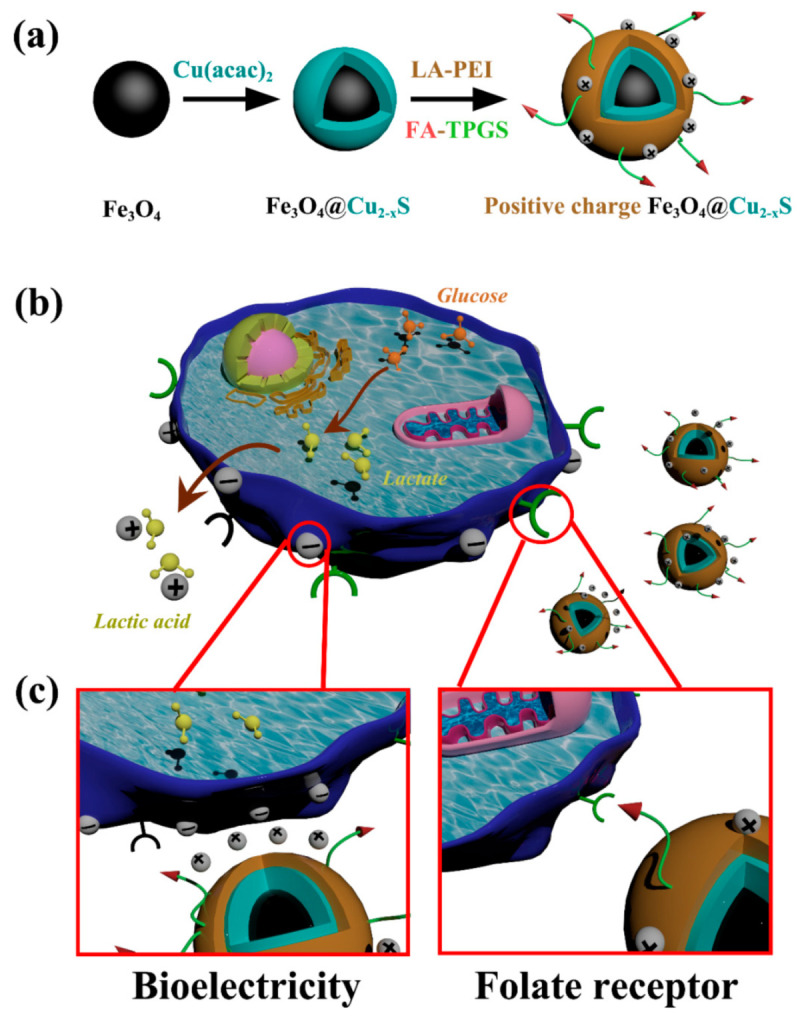
(**a**) Pathway for preparation of the nanoparticles; (**b**) schematic illustration of the Warburg effect, and (**c**) the strategies for electrical charge- and biomarker-mediated cancer targeting (passive and active targeting) via nanoparticles.

The novel concept is schematically depicted in Figure 1. As shown in this figure, a cationic amphiphilic polymer: lauric acid–polyethylenimine (LA-PEI) is coated on the Fe_3_O_4_@Cu_2–x_S nanoparticles and stabilized by the D-α-tocopheryl polyethylene glycol succinate (Vitamin E TPGS or TPGS) to form the positively-charged nanoparticles: PEI-Fe_3_O_4_@Cu_2–x_S. TPGS is a widely used adjuvant in drug delivery which has been approved by the FDA [38]. This biocompatible amphiphilic molecule can be used as a surface stabilizer for enhanced drug stability [38,39,40]. Furthermore, the surfaces of the Fe_3_O_4_@Cu_2–x_S nanoparticles are conjugated with the folic acid (FA-PEI-Fe_3_O_4_@Cu_2–x_S) to increase the targeting efficiency on the folate receptor-expressing cell lines.

Figure 1a shows the schematic pathway for preparation of the positively charged Fe_3_O_4_@Cu_2–x_S nanoparticles. The Fe_3_O_4_ nanoparticles are synthesized through a thermal decomposition process. The as-synthesized Fe_3_O_4_ nanoparticles are then coated with a Cu_2–x_S layer. The hydrophobic Fe_3_O_4_ or Fe_3_O_4_@Cu_2–x_S are stabilized with the amphiphilic polymers and TPGS in order to transfer the nanoparticles from organic solvent to an aqueous phase. Figure 1b illustrates the Warburg effect in cancer cells. As shown in this figure, the negative surface charges are created due to secretion of lactic acid by cancer cells. Figure 1c depicts the concept of the dual-targeting via both surface charges and biomarkers. PEI, as a cationic polymer, provides the positive charges on the nanoparticle surfaces enabling their binding onto the negatively-charged cancer cells. The folic acid modified on TPGS further increases the interaction between the nanoparticles and cancer cells with the folate receptor overexpression. 

## 2. Materials and Methods

### 2.1. General 

All chemicals for nanoparticle synthesis, including iron (III) acetylacetonate (Fe (acac)_3_, ≥99.9%), copper (II) acetylacetonate (Cu (acac)_2_, ≥99.9%), oleylamine (70%), sulfur (99.998%), N-methyl-2-pyrrolidone (99.5%), polyethylenimine (PEI, branched, Mw 600), D-α-tocopheryl polyethylene glycol succinate (Vitamin E TPGS or TPGS), polymer(isobutylene-alt- maleic anhydride) (Mw = 6000), Hexadecylamine (98%), folic acid (FA, ≥97%), and 4-dimethylaminopyridine (DMAP), were purchased from Sigma-Aldrich Inc. (St. Louis, MO, USA). 1-Ethyl-3-(3-dimethylaminopropyl) carbodiimide hydrochloride (EDC), N-Hydroxysuccinimide (NHS) lauric acid (LA), and the organic solvents including chloroform, cyclohexane, and tetrahydrofuran, were purchased from Fisher Scientific Inc. (Hampton, NH, USA). 

The cell culture materials, including RPMI-1640 medium, Dulbecco’s Modified Eagle’s medium (DMEM), fetal bovine serum (FBS), penicillin-streptomycin and 0.25% trypsin-EDTA, were purchased from Thermo Fisher Scientific, Waltham, MA, USA. Dulbecco’s Phosphate Buffered Saline (DPBS), and phosphate-buffered saline (PBS) were purchased from Corning Corp, Corning, NY, USA. Cell Counting Kit-8 (CCK-8), Cyanine5 NHS ester, Cyanine5 amine (non-sulfonated), Calcein-AM, and Propidium Iodide (PI) were purchased from Apexbio Technology LLC (Houston, TX, USA).

### 2.2. Synthesis of Fe_3_O_4_ and Fe_3_O_4_@Cu_2–x_S Nanoparticles

Fe_3_O_4_ and Fe_3_O_4_@Cu_2–x_S nanoparticles were synthesized as described in previous studies with modification [16,17]. Briefly, a certain amount of Fe (acac)_3_ in the NMP/oleylamine mixture (4:3, *v*/*v*) was injected into a preheated oleylamine at 300 °C under a nitrogen protection. Keeping the system at 300 °C for 10 min with stirring, it was cooled down to 60 °C for collecting the Fe_3_O_4_ nanoparticle which was washed by methanol. The dried Fe_3_O_4_ nanoparticle was dispersed in the chloroform until use.

For Cu_2–x_S coating, a certain amount of sulfur solution in an oleylamine/cyclohexane mixture (6:5, *v*/*v*) was injected into the as-synthesized Fe_3_O_4_ nanoparticle at 70 °C. Subsequently, Cu (acac)_2_ was dissolved in an oleylamine/chloroform mixture (1:4, *v*/*v*). This very mixture was then injected into the reaction system and kept at 70 °C for 0.5 h with stirring to obtain the Fe_3_O_4_@Cu_2–x_S nanoparticles. The collected Fe_3_O_4_@Cu_2–x_S nanoparticles were washed with methanol and the dried Fe_3_O_4_@Cu_2–x_S nanoparticles were dispersed in chloroform until use.

### 2.3. LA-PEI and Folate Modified TPGS Synthesis

The amphiphilic PEI was developed from modified hydrophilic fatty acid molecules via EDC/NHS coupling, as described previously [41,42,43,44]. Briefly, the molar ratios of EDC to LA and NHS to EDC were respectively set at 1.25:1 and 1.25:1. They were mixed in the ethanol with 10% MES buffer (100 mM, pH = 6). After 15 min of reaction at 40 °C, solvated PEI (0.25 eqv. molar to lauric acid) was quickly added into the solution and allowed to react for 24 h at 40 °C. The product of LA-PEI was purified by dialysis for three days. 

The synthesis of the folate-modified TPGS followed a modified procedure from a previously reported method [45]. FA, CDI, and DMAP (with molar ratio of 1:1.2:0.5) were dissolved in DMSO (with a FA concentration of 20 mg/mL) and stirred at room temperature for 24 h. TPGS (1 eqv. molar to FA) was then added to the reaction system for another 24 h. The product of FA-TPGS was purified by dialysis (MWCO 1 kDa) for three days. 

### 2.4. Polymer Coating of the Fe_3_O_4_ and Fe_3_O_4_@Cu_2–x_S Nanoparticles

The hydrophobic Fe_3_O_4_ and Fe_3_O_4_@Cu_2–x_S nanoparticles were transferred from organic to aqueous solution by coating the amphiphilic polymers onto the particle surfaces [46]. For the positively-charge nanoparticles, the Fe_3_O_4_ or Fe_3_O_4_@Cu_2–x_S nanoparticles were dissolved in chloroform (1 mL) and added to deionized water (10 mL) containing LA-PEI and FA-TPGS. After sonication for 30 min, the chloroform in the oil-in-water emulsion was evaporated. The extra polymers were removed by dialysis for 48 h. For comparison, the amphiphilic polymer coating on the negatively charged nanoparticles was developed according to the previous reports [16,47,48]. Subsequently, 272 mg of polymer (isobutylene-alt-maleic anhydride) and 320 mg of hexadecylamine were dissolved in THF and heated to 60 °C. It was kept at 60 °C until a cloudy mixture became transparent and all THF had been evaporated. The resulting polymer was dissolved again in anhydrous chloroform. For the negatively charged polymer coating, the Fe_3_O_4_ or Fe_3_O_4_@Cu_2–x_S nanoparticles were mixed with TPGS in chloroform at a mass ratio of 5 to 2 under sonication for a homogeneous mixture. The mixture was then added into a polymer solution (100 mg/mL in chloroform) with negative charges and ultrasonicated for another 5 min. Upon rotary evaporation of the organic solvent, the nanoparticles were dissolved in an aqueous sodium borate buffer (SBB, pH 12) and ultrasonicated for 15 min. The extra polymers were removed by dialysis and the final products were kept at 4 °C until use. For the Cy5 fluorescence dye labeled nanoparticles, the Cy5-NHS ester or Cy5-amine were added to the PEI nanoparticles or to the EDC-NHS negatively charge polymer coated nanoparticles at a mass ratio of 1:100 (dye to nanoparticle). The extra unreacted fluorescence dye was removed over three days of dialysis (MWCO 20 kDa).

### 2.5. Nanoparticle Characterizations

The hydrodynamic diameter and surface potential were determined by dynamic light scattering (DLS) using a Zetasizer Nano-ZS (Malvern, Malvern, UK). For the photothermal experiments, samples were irradiated by using an 808 nm laser (Q-BAIHE, Shenzhen, China) with power of 2 W/cm^2^. The temperature was measured and recorded by using an infrared camera (FLIR E6). The power density of the solar simulator was calibrated by an optical power meter (1919-R, Newport Corporation, Irvine, CA, USA). Nanoparticle size was determined by transmission electron microscopy (CM-20 TEM). The absorption and transmittance spectra were obtained by using a UV–VIS NIR spectrometer Lambda 900 (PerkinElmer Inc., Waltham, MA, USA). The X-ray diffraction analysis was acquired by X-ray Diffractometer (X’Pert MPD).

### 2.6. Cell Lines and Culture Conditions

Three cancer cell lines were used in these studies: RD 769 Mouse Rhabdomyosarcoma, A549 Human Lung Adenocarcinoma, and MDA-MB-231 Human Breast Carcinoma. The non-malignant CCD-19Lu Human Lung Fibroblast Cell Line was used for comparison with cancer cells. The A549 and CCD-19Lu cell lines were purchased from American Type Culture Collection (ATCC). The RD 769 rhabdomyosarcoma cell line was a kind gift from Dr. Timothy Cripe (Nationwide Children’s Hospital, OH). The MDA-MB-231 breast cancer cell line was a kind gift from Dr. Jun-Lin Guan (University of Cincinnati, Cincinnati, OH, USA). The RD 769 and MDA-MB-231 cell lines were cultured in Dulbecco’s modified Eagle’s medium (DMEM). The A549 cell line was cultured in Kaighn’s Modification of Ham’s F-12 Medium (F12K), and the CCD-19Lu cell line was cultured in Eagle’s Minimum Essential Medium (EMEM). All mediums were supplemented with 10% fetal bovine serum (FBS) and 1% antibiotic–antimycotic. Cells were maintained at 37 °C in 5% CO_2_ humidified atmosphere.

### 2.7. Calcein-AM/PI Assay

After photothermal treatment, a mixture of Calcein-AM (4 μM) and Propidium Iodide (8 μM) in DPBS was added to the cells and incubated for 10 min for co-staining. The live cells were labeled in green color by Calcein-AM and the dead cells were labeled in red color by PI. The EVOS M7000 fluorescence microscope was used to examine the live/dead cells.

### 2.8. Confocal Microscopy Imaging

The cells were seeded in 8 chamber-slides 24 h before the experiments. Nanoparticles labeled with Cy5 were incubated with the cells at 37 °C and the excess of NPs was removed by washing with PBS. The cells were fixed using 4% PFA (Paraformaldehyde, Electron Microscopy Sciences, Hatfield, PA, USA), and the cell nuclei were counterstained with DAPI (4′,6-diamidino-2-phenylindole, blue). The cells and nanoparticles were prepared, imaged and analyzed using a Nikon A1R GaAsP inverted confocal microscope as described [49,50].

### 2.9. Flow Cytometry Analysis

The cells were seeded in 24-well plates and incubated for 24 h before the flow cytometry analysis. The nanoparticles were added to the cells and incubated at 37 °C for 5 min. The cells were subsequently rinsed with PBS, trypsinized, and transferred to tubes. Cell-associated fluorescence was determined using a BD LSR II flow cytometer, and the data were analyzed using the FlowJo software, as previously described [51,52]. 

### 2.10. Real-Time Quantitative RT-PCR (qRT-PCR)

Total RNA was extracted from cultured cells using the RN easy micro-Kit (Qiagen, Germantown, MD, USA) as described [53,54]. The cDNA was generated using iScript cDNA Synthesis Kits (BIO-RAD, Hercules, CA, USA). Quantitative real-time RT-PCR (qRT-PCR) was performed according to the TaqMan Gene Expression Assay protocols (Invitrogen, Waltham, CA, USA) [55,56]. 

### 2.11. Photothermal Conversion Efficiency

The photothermal conversion efficiencies (*η*) of Fe_3_O_4_ and Fe_3_O_4_@Cu_2–x_S nanoparticles were calculated using the equations developed by Roper et al. [19,57]. The photothermal conversion efficiency can be expressed by the following:

(1) $$\eta =\frac{hS\left(T_{Max}–T_{sur}\right)\;–Q_{s}}{I\left(1\;–10^{–A_{808}}\right)}$$

where *h* is the heat transfer coefficient (W × m^−2^×°C^−1^); *S* is the surface area of the container (m^2^); *T_Max_* is the maximum temperature of the solution (°C); *T_Sur_* is the surrounding temperature; *Q_s_* is the energy input by the sample cuvette and the solution (W), *I* is the incident laser power (W), and *A*_808_ is the absorbance of the Fe_3_O_4_ and Fe_3_O_4_@Cu_2–x_S nanoparticles in the standard rectangular glass cell with lid at the wavelength of 808 nm. 

The value of *hS* is obtained by the following equation [16,19,57]:

(2) $$hS=\frac{m_{H_{2}O}C_{P,\;H_{2}O}}{\tau_{S}}$$

where $m_{H_{2}O}$ and $C_{P,\;H_{2}O}$ are respectively the mass (g) and heat capacity (J/g×°C) of the sample. *τs* is the sample system time (s) which is given by [57]:

(3) $$\tau_{S}=–\frac{t}{\ln\theta }$$

where *θ* is defined as the ratio of (*T − T_Sur_*) to (*T_Max_ − T_Sur_*), and *T* is the solution temperature (°C). In this research, the heat capacity of water is 4.18 J/g, the mass of the solution is 0.1 g, the incident laser power is 0.5 W, *A*_808_ was determined to be 0.53071, and *Q_s_* is 0.005 W.

### 2.12. In Vitro Photothermal Cancer Killing Efficiency

The in vitro cancer cell killing efficiency was assessed by using the Cell Counting Kit (CCK-8, Apexbio Technology LLC). The cells were seeded on 96-well plates 24 h prior to the photothermal experiments. The nanoparticles were diluted to different concentrations in the DPBS and incubated with the cells for 5 min at 37 °C. Excess NPs were removed and replaced by PBS. The cells were irradiated with an 808 nm laser (2 W cm^−2^) for 5 min. After that, 10 μL CCK-8 was added to the plate and incubated at 37 °C for 3 h. The assay absorbance was measured at a wavelength of 450 nm in a Microplate Reader. The viability of the cell was calculated by the equations below:

Cell viability (%) = [(As − Ab)/(Ac − Ab)] × 100

As = Absorbance of treated cell
Ac = Absorbance of untreated cell
Ab = Absorbance of blank background

## 3. Results and Discussion

**Figure 2:**
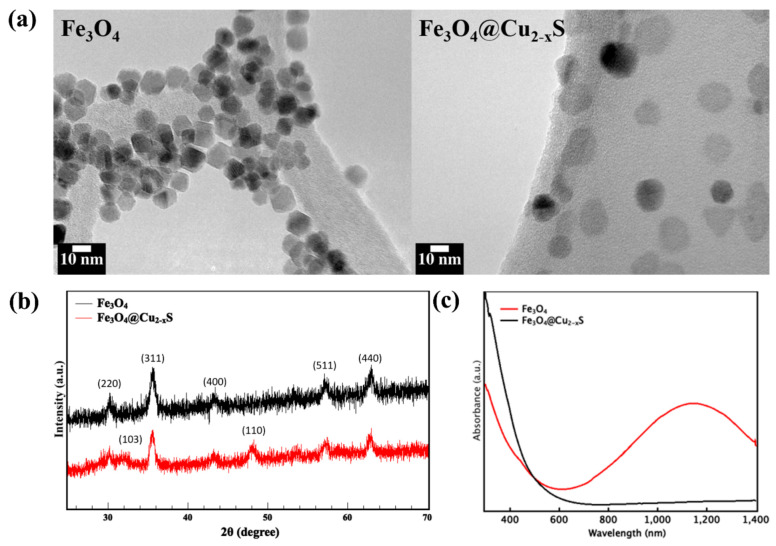
(**a**) TEM images of the Fe_3_O_4_ and Fe_3_O_4_@Cu_2–x_S nanoparticles; (**b**) powder X-ray diffraction patterns of the Fe_3_O_4_ and Fe_3_O_4_@Cu_2–x_S nanoparticles, and (**c**) UV−vis NIR absorption spectra for solutions of the Fe_3_O_4_ and Fe_3_O_4_@Cu_2–x_S nanoparticles dispersed in toluene.

Figure 2a shows the TEM images of the Fe_3_O_4_ and Fe_3_O_4_@Cu_2–x_S nanoparticles without surface modification. The average sizes of the Fe_3_O_4_ and Fe_3_O_4_@Cu_2–x_S nanoparticles were ~10 nm and ~15 nm, respectively. The X-ray powder diffraction patterns of both Fe_3_O_4_ and Fe_3_O_4_@Cu_2–x_S are shown in Figure 2b. As can be seen in this figure, all diffraction peaks can be assigned to Fe_3_O_4_ with the crystal planes identified. In addition, the peaks of the (103) and (110) planes are identified for CuS respectively at 2𝜃 = 31.8° and 48.1°. 

The UV−vis NIR absorption spectra of Fe_3_O_4_ and Fe_3_O_4_@Cu_2–x_S solutions are shown in Figure 2c. As shown in this figure, Fe_3_O_4_ is characterized by a strong UV absorption, but no peak is observed in the IR region. With a Cu_2–x_S shell on Fe_3_O_4_, however, there is a pronounced IR absorption at 1160 nm in Fe_3_O_4_@Cu_2–x_S. The enhanced IR absorption can be utilized for creating strong photothermal effects in PTT.

The amphiphilic polymer coating was designed to stabilize the nanoparticles in aqueous solutions and control the surface functionalization, including surface charge and targeting ligands. Vitamin E TPGS-Folic acid (FA-TPGS) (Figure S1a) and lauric acid-Polyethylenimine (LA-PEI) (Figure S1b) were synthesized for nanoparticle surface modifications. The cationic polymer PEI provides the positive surface charges for the nanoparticles, combined with folic acid modification to enhance the nanoparticles’ interactions with cancer cells efficiently. The structures of the amphiphilic polymers were characterized with ^1^H-NMR (Figure S2). The ^1^H-NMR of FA shows a peak in the region around 12 ppm corresponding to the proton signal of the carboxyl groups of FA (Figure S2a) which is not seen in the ^1^H-NMR FA-TPGS (Figure S2c). Similarly, the proton signal of the carboxyl groups of LA (Figure S2d) is also not shown in the reaction products (Figure S2f). These results indicate that the carboxyl group on FA and LA was successfully coupled to TPGS and PEI, respectively. Other characteristic peaks in the reactants are present in the final products, indicating successful synthesis of FA-TPGS (Figure S2c) and LA-PEI (Figure S2f).

**Figure 3:**
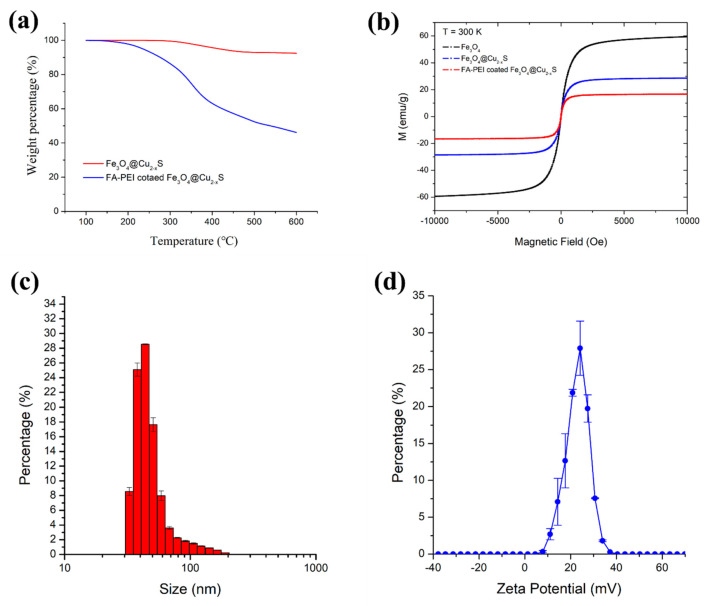
(**a**) Thermogravimetric analysis (TGA) curves of uncoated Fe_3_O_4_@Cu_2–x_S and FA-PEI- Fe_3_O_4_@Cu_2–x_S; (**b**) magnetic hysteresis loops of Fe_3_O_4_, Fe_3_O_4_@Cu_2–x_S, and FA-PEI- Fe_3_O_4_@Cu_2–x_S; (**c**) size distribution of FA-PEI- Fe_3_O_4_@Cu_2–x_S nanoparticle, and (**d**) surface zeta potential of FA-PEI- Fe_3_O_4_@Cu_2–x_S.

Thermogravimetric analysis (TGA) was used to quantify the polymer coating on the Fe_3_O_4_@Cu_2–x_S nanoparticles. As shown in Figure 3a, the total weight loss of FA-PEI-Fe_3_O_4_@Cu_2–x_S is 7.55%. With surface modification, the weight loss in temperatures ranging from 200 °C to 450 °C increases to 42.76%. The increase in weight loss in this region is due to the decomposition of the amphiphilic polymers coating. Further weight loss from 450 °C to 600 °C is from the char residue, which is 11.1%. The TGA data demonstrate the mass ratio of the polymer coating to Fe_3_O_4_@Cu_2–x_S to be approximately 1:1. 

The magnetic properties of the Fe_3_O_4_, Fe_3_O_4_@Cu_2–x_S, and coated Fe_3_O_4_@Cu_2–x_S were characterized by vibrating-sample magnetometer (VSM). The magnetic hysteresis curves are shown in Figure 3b. As shown in this figure, the saturation magnetization of Fe_3_O_4_ is 59. 4 emu/g, considerably larger than that of Fe_3_O_4_@Cu_2–x_S (28.6 emu/g). The reduction in magnetization in Fe_3_O_4_@Cu_2–x_S is attributed to the non-magnetic Cu_2–x_S component on the surfaces of the Fe_3_O_4_ nanoparticles. The saturation magnetization is reduced to 16.67 emu/g after coating Fe_3_O_4_@Cu_2–x_S nanoparticles with non-magnetic polymers. However, all nanoparticles show superparamagnetic behavior reflected by the highly reversible hysteresis curves regardless of the surface modifications by Cu_2–x_S or polymer coating. 

The hydrodynamic size and the surface charge were determined using Dynamic light scattering (DLS). The size distribution of the FA-PEI- Fe_3_O_4_@Cu_2–x_S nanoparticles is majorly in the range of 37–50 nm (Figure 3c) with a surface charge of 27.33 ± 0.69 mV (Figure 3d). Upon surface modification, the average hydrodynamic diameter extends to 192.37 ± 2.15 nm, which is in an appropriate average range (180–220 nm) for accumulating readily in tumor vasculature as compared to those in the previously reported studies on medical diagnosis and therapeutics [10,40,58,59,60,61]. For comparison, the negatively-charged nanoparticles were used as control in the photothermal cancer cell killing experiments, following the procedures reported previously [16,47,48]. The size and zeta potential distributions of the negatively-charged nanoparticles are shown in Figure S3a,b. The average hydrodynamic diameter and the surface charge of the negatively charged nanoparticles are 142.27 ± 2.71 nm and −31.5 ± 1.23 mV, respectively. The polymer weight percentage of the negatively-charged nanoparticles (determined by TGA) is 55.33% (Figure S3c), which is 12.57% higher than that of the FA-PEI-Fe_3_O_4_@Cu_2–x_S nanoparticles. 

**Figure 4:**
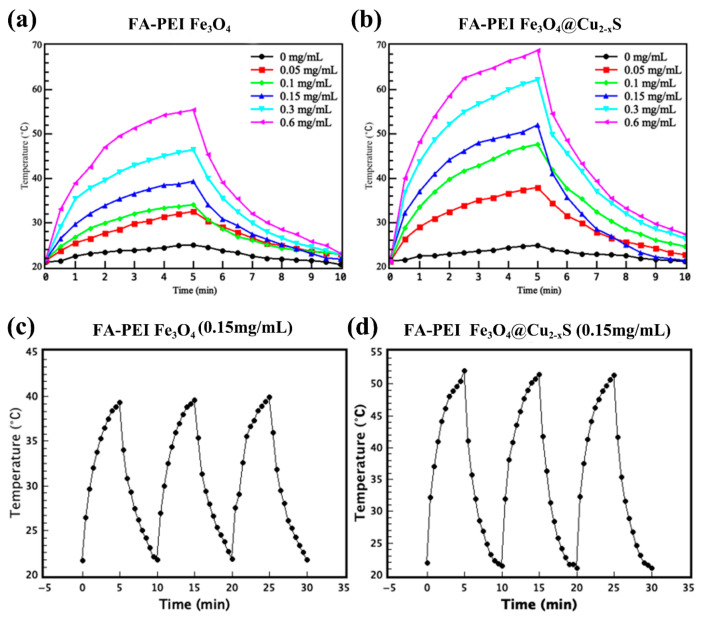
Temperature vs. time curves of (**a**) FA-PEI- Fe_3_O_4_ and (**b**) FA-PEI- Fe_3_O_4_@Cu_2–x_S at different concentrations. The temperature vs. time curves of (**c**) FA-PEI- Fe_3_O_4_ and (**d**) FA-PEI- Fe_3_O_4_@Cu_2–x_S for three on/off cycles.

Figure 4a,b show the photothermal properties of the FA-PEI functionalized Fe_3_O_4_ and Fe_3_O_4_@Cu_2–x_S nanoparticles with varied concentrations under 808-nm laser irradiation (2 W/cm^2^). The nanoparticles were dispersed in water and placed in a 96-well plate (100 uL aqueous solution). After the solution was irradiated for five minutes, the light source was turned off, and the temperatures were measured by infrared thermal camera. Figure 4a,b show, respectively, the temperature increases as function of time for the surface-functionalized Fe_3_O_4_ (Figure 4a) and Fe_3_O_4_@Cu_2–x_S (Figure 4b) nanoparticles of various concentrations irradiated by 808 nm laser (2 W/cm^2^). As shown in these figures, the temperature increases for both particle systems at the beginning are rather rapid due to the photothermal effects of the nanoparticles, but leveling off after 1 min as a result of heat loss through the environment. The light is turned off at 5 min and temperatures are thereafter decreasing rapidly for all concentrations. As can also be seen in these figures, the heating curves are consistent with the particle concentrations that the highest temperature reaches 56 °C for FA-PEI- Fe_3_O_4_ at the concentration of 0.6 g/mL and 68 °C for FA-PEI- Fe_3_O_4_@Cu_2–x_S at the same concentration. This significant increase in temperature in the latter is due to the pronounced IR absorbance in the Fe_3_O_4_@Cu_2–x_S solution. Therefore, the Fe_3_O_4_@Cu_2–x_S nanoparticles are expected to exert a much stronger photothermal effect than that of the Fe_3_O_4_ counterpart. 

The photostability of the functionalized Fe_3_O_4_ and Fe_3_O_4_@Cu_2–x_S nanoparticles were characterized by three on/off cycles of laser irradiation (Figure 4c,d). By turning light off and on every 10 min, the heating curves show consistent increases and decreases after several cycles indicating good photostability of the nanoparticles. The negatively-charged Fe_3_O_4_@Cu_2–x_S nanoparticles also show similar photothermal effects and photostabilities as shown in Figure S3d,e. However, the negatively-charged nanoparticles show weaker photothermal effect due to its higher polymer to particle ratio in comparison to the positively-charged FA-PEI- Fe_3_O_4_@Cu_2–x_S at the same concentration. The photothermal conversion efficiency at 0.15 mg/mL is calculated by Equations (1)–(3) and the results are shown in Table S1.

**Figure 5:**
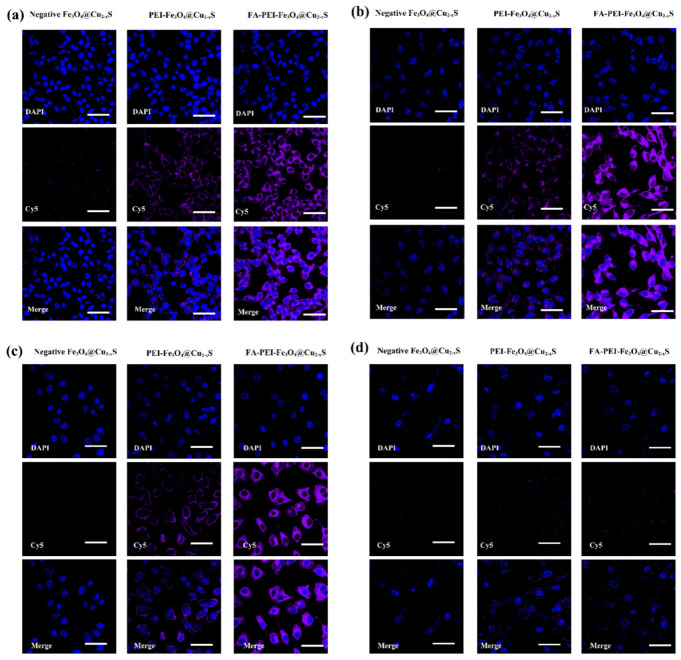
Cy5 labeled nanoparticle binding to the cancer cells. (**a**) RD 769, (**b**) MDA-MB-231, (**c**) A549, and (**d**) CCD-19Lu. DAPI was used to stain the cell nucleus and Cy5 was labeled on the nanoparticles. Scale bar, 50 µm.

The cancer cell binding efficiencies of different nanoparticles were assessed by incubating the cells with the Cy5-fluorescent-dye-labeled nanoparticles for 5 min, and extra nanoparticles were removed by washing with PBS. The Fe_3_O_4_@Cu_2–x_S nanoparticles with the negative-charged polymer coating were used as comparison. Compared with the negative Fe_3_O_4_@Cu_2–x_S nanoparticle treated cancer cells (which hardly displays any Cy5 signal), the positive PEI-Fe_3_O_4_@Cu_2–x_S treated cancer cells show visible Cy5 signals around the cancer cells (Figure 5a–c). With FA functionalization, the FA-PEI Fe_3_O_4_@Cu_2–x_S nanoparticles show a significant fluorescence increase on the cancer cells (Figure 5a–c). The normal cell line (CCD-19Lu) interacted with neither the negative, nor the positive or folate modified positive nanoparticles due to their neutral surfaces. Therefore, the Cy5 signal from all three nanoparticles is not observed on CCD-19Lu cells (Figure 5d). These experimental results indicate strong electrostatic interactions between the charged nanoparticles and the cancer cells.

**Figure 6:**
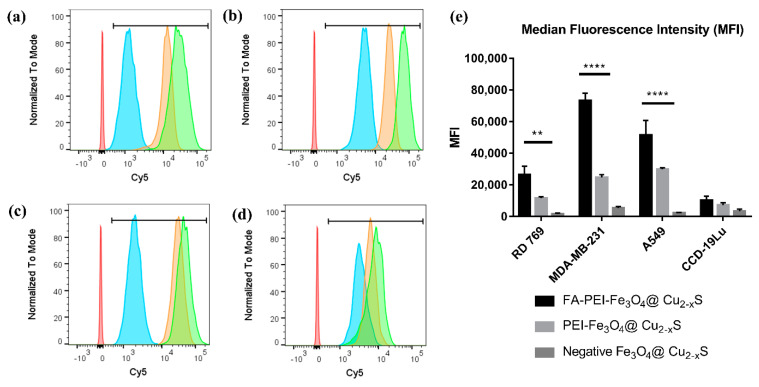
Flow cytometry histogram profiles of (**a**) RD 769 cell line; (**b**) MDA-MB-231 cell line; (**c**) A549 cell line; (**d**) CCD-19Lu cell line with different nanoparticles: negatively charged Fe_3_O_4_@Cu_2–x_S (blue), PEI- Fe_3_O_4_@Cu_2–x_S (orange) and FA-PEI- Fe_3_O_4_@Cu_2–x_S (green), and (**e**) median fluorescence intensities (MFI) of the RD 769, MDA-MB-231, and A549 cell lines. (** *p* < 0.01, **** *p* < 0.0001).

The quantification of the nanoparticle binding to the cancer cells was determined by flow cytometry. As shown in Figure 6a–c, the positively-charged nanoparticles exhibit higher Cy5 fluorescence compared to the negatively-charged Fe_3_O_4_@Cu_2–x_S nanoparticles in all three cancer cell lines. Figure 6d shows the quantification of the median fluorescence intensity (MFI). Compared to the negatively-charged Fe_3_O_4_@Cu_2–x_S nanoparticles, the intensities of Cy5 fluorescence from the positively-charged Fe_3_O_4_@Cu_2–x_S nanoparticles bound onto RD 769, MDA-MB-231 and A459 cells are 7.08-fold, 4.57-fold, and 13.18-fold, respectively (Figure 5e). After FA modification, the Cy5 signals from all three cancer cell lines are further increased to 2.26-fold, 2.97-fold, and 1.72-fold respectively, compared to treatment with the positively-charged nanoparticles without folic acid modification (Figure 5e). The binding efficiency of FA-modified nanoparticles is dependent on folate receptor levels in cancer cell lines. The folate receptor (FOLR1) expression levels in the human cell lines were examined by RT-qPCR and shown in Figure S4. The FOLR1 expression levels in MDA-MB-231 and A549 are, respectively, 11.31-fold and 2.26-fold higher than CCD-19Lu. The highest FOLR1 level of MDA-MB-231 significantly improved the binding efficiency with FA-PEI-Fe_3_O_4_@Cu_2–x_S.

In normal CCD-19Lu cells, the nanoparticles with the positively charged surfaces and folic acid modification only increased the cell binding slightly. Cell binding efficiencies of both PEI-Fe_3_O_4_@Cu_2–x_S and FA-PEI-Fe_3_O_4_@Cu_2–x_S were found to be insignificant when compared with the negatively-charged Fe_3_O_4_@Cu_2–x_S (Figure 5e). The flow cytometry results indicate that the mouse cancer cells have less nanoparticle binding than human cell lines (Figure 5e). However, the positively-charged surfaces and folic acid modification still increased the targeting efficiency. In human cancer cell lines, the positively-charged nanoparticle binding efficiencies on cancer cell lines are 3.37-fold for MDA-MB-231 and 4.08-fold for A549, both are higher than that on the normal cell line (CCD-19Lu) (Figure 5e). Upon folic acid modification, the nanoparticle binding efficiencies on cancer cell lines are further increased to 7.15-fold (MDA-MB-231) and 5.02-fold (A549) compared with the normal cell line (CCD-19Lu) (Figure 5e). Fluorescent microscopy and flow cytometry data demonstrate that the FA-PEI-Fe_3_O_4_@Cu_2–x_S has high targeting efficiency to the cancer cell lines, as shown in Figure 5e.

**Figure 7:**
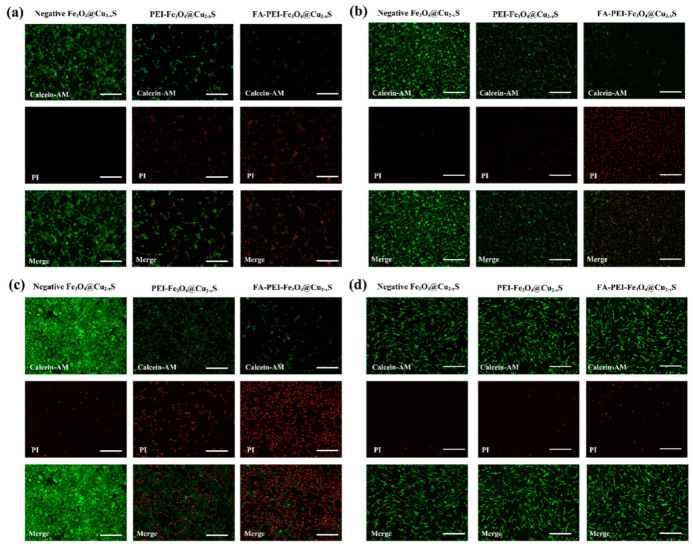
In vitro photothermal therapy effect of the negatively-charged Fe_3_O_4_@Cu_2–x_S, positively-charged PEI- Fe_3_O_4_@Cu_2–x_S, and FA-PEI- Fe_3_O_4_@Cu_2–x_S at 0.32 mg/mL on (**a**) RD 769 cell line; (**b**) MDA-MB-231 cell line; (**c**) A549 cell line, and (**d**) CCD-19Lu cell line with 5 min 2 W cm^−2^ 808-nm laser irradiation. Calcein-AM/PI live-dead staining was used to stain live (green) and dead (red) cells. Scale bar, 275 μm.

The Calcein-AM/PI live-dead staining is shown in Figure 7. Under the same conditions (5 min incubation, 2 W cm^−2^ 808 nm laser irradiation for 5 min), the negatively-charged Fe_3_O_4_@Cu_2–x_S with laser irradiation did not cause significant cell death in all three cancer cell lines. After treatment with positively-charged PEI-Fe_3_O_4_@Cu_2–x_S nanoparticles, however, the cancer cells showed higher red fluorescence intensity, indicating effective cancer cell killing by the photothermal effect. The folic acid modification further increased the cancer cell killing efficiency due to higher nanoparticle-cancer cell binding efficiency. Figure 7a shows the toxicities of different nanoparticles (0.32 mg/mL) incubated with RD 769 (Figure S5a), MDA-MB-231 (Figure S5b), A549 tumor cells (Figure S5c), and normal CCD-19Lu cells (Figure S5d) without laser irradiation. As shown in Figure 7, with folic acid modification, the nanoparticle toxicities to the cancer cells are higher than those without FA modification (Figure 6a–c), due to the higher binding efficiency of FA-PEI-Fe_3_O_4_@Cu_2–x_S. However, the cell death caused by the FA-PEI-Fe_3_O_4_@Cu_2–x_S nanoparticles without laser treatment is significantly lower than that with laser, indicating strong photothermal effect of FA-PEI-Fe_3_O_4_@Cu_2–x_S. For normal CCD-19Lu cells, none of the nanoparticles exhibited high toxicity, with or without laser (Figure 6d and Figure S5d) due to insignificant particle binding on normal cells since they are practically neutral compared to the negatively charged cancer cells. The 808-nm laser irradiation treatment without nanoparticles did not show significant cytotoxicity for either cancer or normal cell lines (Figure S5e).

**Figure 8:**
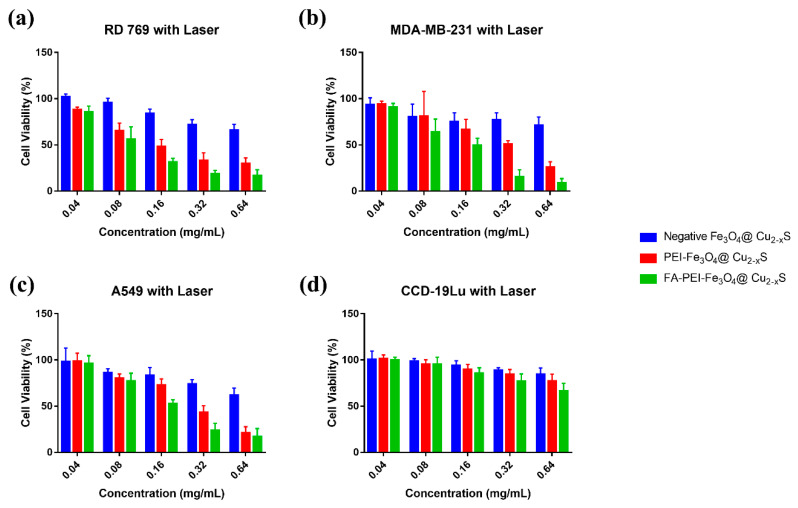
Cell viability vs. concentration of different nanoparticles for cancer cell lines (**a**) RD 769; (**b**) MDA-MB-231; (**c**) A549 and a normal cell line, and (**d**) CCD-19Lu under photothermal treatments (2 W cm^−2^ 808-nm laser irradiation for 5 min).

The cell viabilities at different nanoparticle concentrations are shown in Figure 8. As shown in Figure 8a–c, with laser treatment, the FA-PEI-Fe_3_O_4_@Cu_2–x_S nanoparticles inflict the strongest photothermal cancer cell killing in all three cancer cell lines due to the highest cell binding efficiency, followed by the PEI-Fe_3_O_4_@Cu_2–x_S nanoparticles. In contrast, the cancer cell killing was negligible after photothermal treatment with the negatively-charged Fe_3_O_4_@Cu_2–x_S nanoparticles, likely, due to weak nanoparticle binding on cancer cell surfaces. The viabilities of cancer cells without laser treatment were significantly higher than the laser-treated counterpart groups with the same nanoparticle concentrations (Figure S6a–d). Compared with cancer cells, the normal cells show much higher cell survival rate for all groups. These quantitative data show that the FA-PEI-Fe_3_O_4_@Cu_2–x_S nanoparticles have much greater cancer cell photothermal killing efficiency with negligible influence on normal cells. 

Based on the in vitro data in this study, we have shown that the nanoparticle surfaces conjugated with both positive electrical charge and folic acid have significantly enhanced cancer cell binding, leading to improved photothermal killing efficiency. The outcomes of this study can be applied to other nano-carrier systems for more effective photothermal therapy. The dual-targeting concept will require future in vivo experiments to demonstrate its validity in preclinical settings.

## 4. Conclusions

We have synthesized both Fe_3_O_4_ and Fe_3_O_4_@Cu_2–x_S nanoparticles and compared their characteristics in optical absorption and photothermal effect for enhanced photothermal cancer therapy. By modifying the particle surfaces of Fe_3_O_4_ with CuS, we have developed the Fe_3_O_4_@Cu_2–x_S nanoparticles that exhibit pronounced IR abortions that contribute to much stronger photothermal effect in cancer cell killing compared to the Fe_3_O_4_ counterparts. As a result, the photothermal conversion efficiency of Fe_3_O_4_@Cu_2–x_S has increased by 29.18%, while that of Fe_3_O_4_ is only 22.99%. Both Fe_3_O_4_ and Fe_3_O_4_@Cu_2–x_S are surface-modified with polymer coatings for dual targeting with cell surface electrical charge and folic acid. As all cancer cell surfaces are negatively charged due to high glycolysis rates, rendering the positively-charged nanoparticles enables efficient binding onto cancer cells for enhanced photothermal cancer cell killing. The cationic polymer coating on the nanoparticles has been found to facilitate the nanoparticle binding to cancer cells rapidly due to charge difference between the nanoparticles (positive) and the cancer cells (negative). The folic acid modification on the charged nanoparticle surfaces has further enhanced the nanoparticle targeting efficiency via folate receptor, which is overexpressed in cancer cells (active targeting). With the unique dual targeting strategy, the FA-PEI-Fe_3_O_4_@Cu_2–x_S nanoparticles show much higher cancer cell binding and subsequent photothermal cancer cell killing without noticeable toxicity to normal cells under the same conditions. In contrast, the negatively-charged Fe_3_O_4_@Cu_2–x_S nanoparticles show insignificant cell binding and photothermal toxicity due to repulsive force between the nanoparticles and cancer cells, since both have the same electrical charge. The experimental results from this study show a promise in photothermal cancer therapy by dual targeting of cancer cells via conjugating both the positive surface charge and the tumor-specific biomarkers on the nanoparticle surfaces. 

## Data Availability

Data available in a publicly accessible repository.

## Supplementary Materials

The following are available online at https://www.mdpi.com/article/10.3390/cancers13215275/s1, Figure S1: The chemical structures and synthesis procedures of (**a**) FA-TPGS and (**b**) LA-PEI. Figure S2: 1H-NMR characterization of the coating polymers (**a**) Folic acid, (**b**) TPGS, (**c**) FA-TPGS in DMSO-d6, (**d**) Lauric acid, and (**e**) PEI, and (**f**) LA-PEI in Chloroform-d. Figure S3: (**a**) Size distribution of the negative Fe_3_O_4_@Cu_2–x_S nanoparticle, (**b**) surface zeta potential of negative Fe_3_O_4_@Cu_2–x_S, (**c**) thermogravimetric analysis (TGA) curves of uncoated Fe_3_O_4_@Cu_2–x_S and negative Fe_3_O_4_@Cu_2–x_S, (**d**) temperature vs. time curves for negative Fe_3_O_4_@Cu_2–x_S, and (**e**) temperature vs. time curves of negative Fe_3_O_4_@Cu_2–x_S for three on/off cycles. Figure S4: FOLR1 expression levels in different cell lines. Human FOLR1 mRNA levels in tumor cells were measured by qRT-PCR and normalized to Beta-Actin mRNA. Figure S5: In vitro toxicity of negatively-charged Fe_3_O_4_@Cu_2–x_S, positively-charged PEI- Fe_3_O_4_@Cu_2–x_S, and FA-PEI- Fe_3_O_4_@Cu_2–x_S at 0.32 mg/mL on (**a**) RD 769 cell line, (**b**) MDA-MB-231 cell line, (**c**) A549 cell line, (**d**) CCD-19Lu cell line without laser, and (**e**) 5 min 2 W cm^−2^ 808 nm laser irradiation treatment for different cells without nanoparticle. Scale bar: 275 μm. Figure S6: Cell viability vs. concentration of different nanoparticles for cancer cells (**a**) RD 769, (**b**) MDA-MB-231, (**c**) A549 and (**d**) non-malignant CCD-19Lu cells without laser treatment. Table S1: Photothermal conversion efficiencies of negative Fe_3_O_4_@Cu_2–x_S, PEI-Fe_3_O_4_@Cu_2–x_S, and FA-PEI-Fe_3_O_4_@Cu_2–x_S at concentration of 0.15 mg/mL.
