# Supplementary material for: Dual Targeting with Cell Surface Electrical Charge and Folic Acid via Superparamagnetic Fe3O4@Cu2–xS for Photothermal Cancer Cell Killing
Source: Cancers (Basel). 2021 Oct 21;13(21):5275. doi: 10.3390/cancers13215275 (PMC8582571; doi:10.3390/cancers13215275)
Supplement: Supplementary file 1 [file cancers-13-05275-s001.zip › cancers-1397736-supplementary.pdf]

Supplemental Materials: Dual Targeting with Cell Surface Electrical Charge and Folic Acid via Superparamagnetic $Fe_3O_4@Cu_{2-x}S$ for Photothermal Cancer Cell Killing

[1] The Materials Science and Engineering Program, College of Engineering and Applied Science, University of Cincinnati, Cincinnati, OH 45221, USA; dengzh@mail.uc.edu (Z.D.); lin2jo@mail.uc.edu (J.L.); websteba@mail.uc.edu (B.W.)

[2] Center for Lung Regenerative Medicine, Cincinnati Children's Hospital Medical Center, 3333 Burnet Avenue, Cincinnati, OH 45229, USA

[3] Division of Pulmonary Biology, Cincinnati Children's Hospital Medical Center, 3333 Burnet Avenue, Cincinnati, OH 45229, USA; tatiana.kalin@cchmc.org

[4] Division of Materials Science and Engineering, Ames Laboratory, Ames, IA 50011, USA; budko@ameslab.gov

[5] Department of Pediatrics, College of Medicine, University of Cincinnati and Cincinnati Children's Hospital Medical Center, 3333 Burnet Avenue, Cincinnati, OH 45229, USA

* Correspondence: Vladimir.Kalinichenko@cchmc.org (V.V.K.); donglu.shi@uc.edu (D.S.)

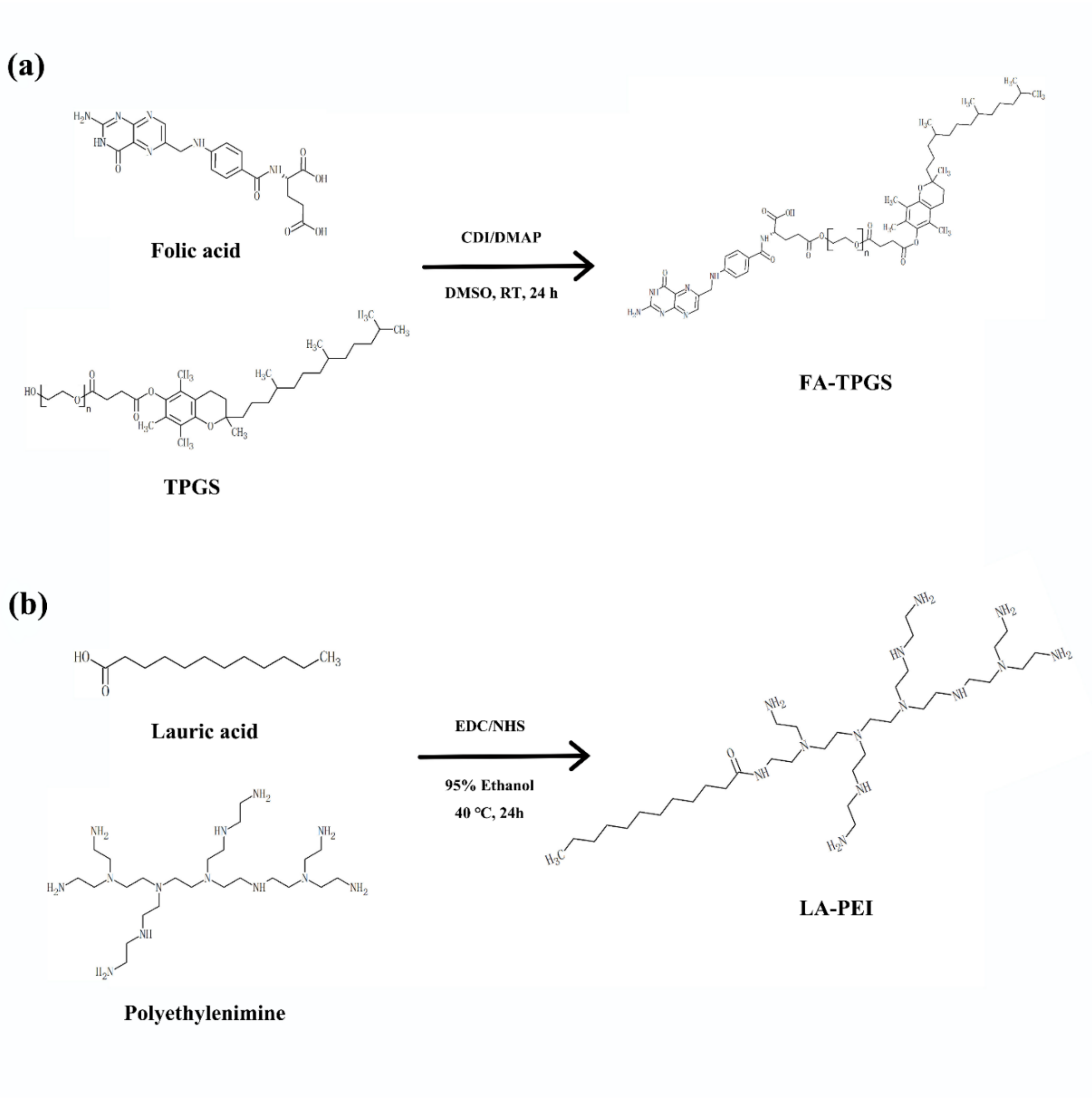

Figure S1. The chemical structures and synthesis procedures of (**a**) FA-TPGS and (**b**) LA-PEI.

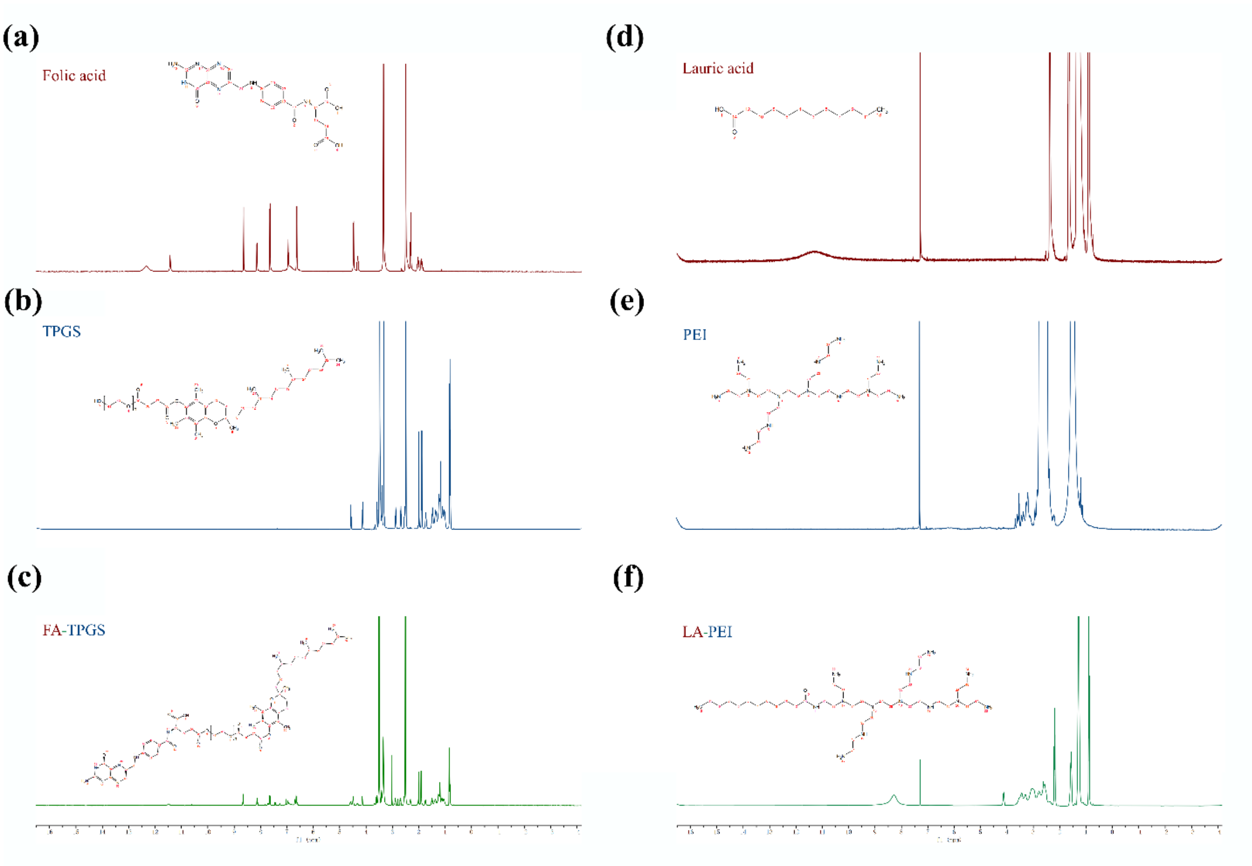

Figure S2. ^{1}H-NMR characterization of the coating polymers (**a**) Folic acid, (**b**) TPGS, (**c**) FA-TPGS in DMSO-d6, (**d**) Lauric acid, and (**e**) PEI, and (**f**) LA-PEI in Chloroform-d.

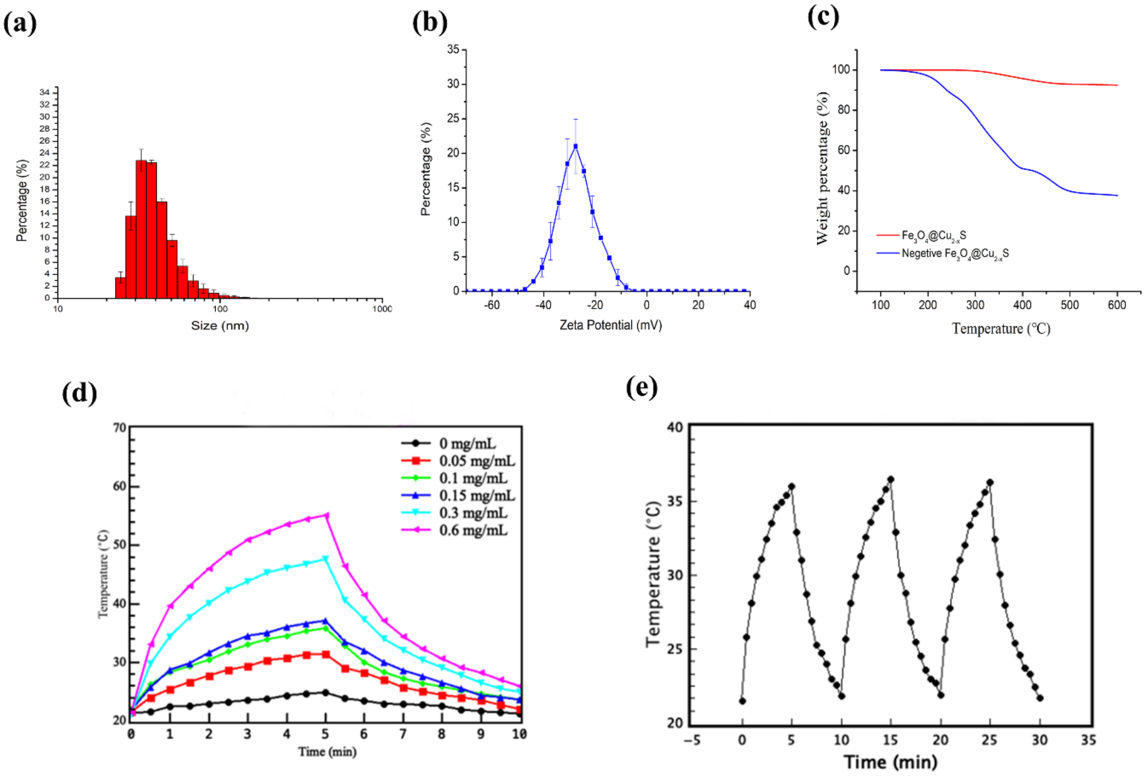

Figure S3. (**a**) Size distribution of the negative $Fe_3O_4@Cu_{2-x}S$ nanoparticle, (**b**) surface zeta potential of negative $Fe_3O_4@Cu_{2-x}S$, (**c**) thermogravimetric analysis (TGA) curves of uncoated $Fe_3O_4@Cu_{2-x}S$ and negative $Fe_3O_4@Cu_{2-x}S$, (**d**) temperature vs.

time curves for negative $Fe_3O_4@Cu_{2-x}S$, and (**e**) temperature vs. time curves of negative $Fe_3O_4@Cu_{2-x}S$ for three on/off cycles.

Table S1. Photothermal conversion efficiencies of negative $Fe_3O_4@Cu_{2-x}S$, PEI-$Fe_3O_4@Cu_{2-x}S$, and FA-PEI-$Fe_3O_4@Cu_{2-x}S$ at concentration of 0.15 mg/mL.

	FA-PEI-Fe_3O_4	Negative $Fe_3O_4@Cu_{2-x}S$	FA-PEI-$Fe_3O_4@Cu_{2-x}S$
η	22.99	16.46	52.17

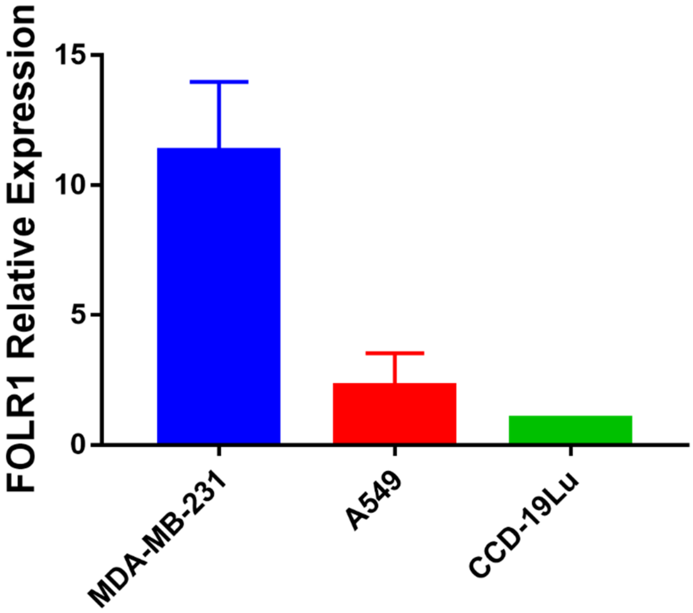

Figure S4. FOLR1 expression levels in different cell lines. Human *FOLR1* mRNA levels in tumor cells were measured by qRT-PCR and normalized to Beta-Actin mRNA.

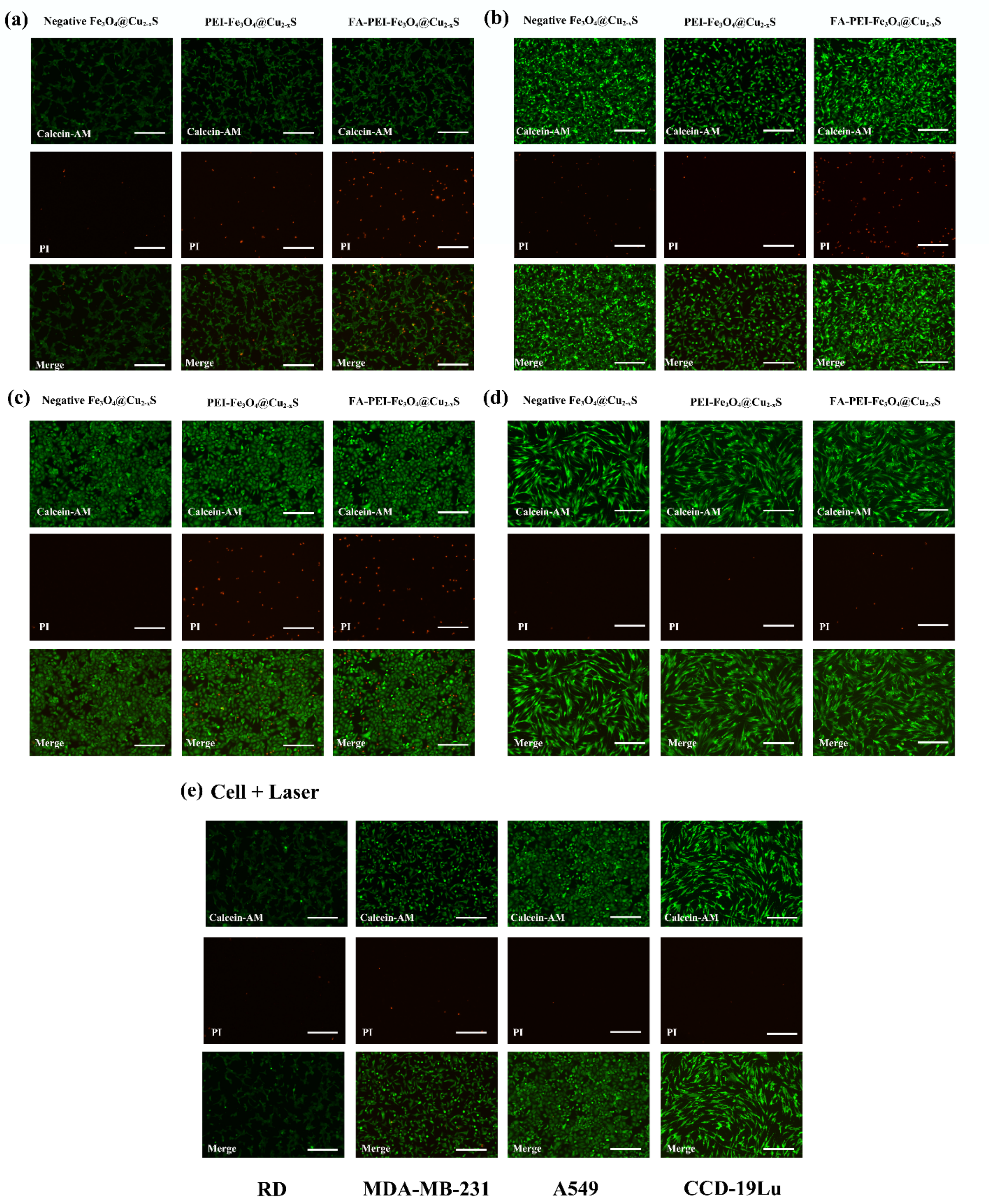

Figure S5. In vitro toxicity of negatively-charged $Fe_3O_4@Cu_{2-x}S$, positively-charged PEI- $Fe_3O_4@Cu_{2-x}S$, and FA-PEI-$Fe_3O_4@Cu_{2-x}S$ at 0.32 mg/mL on (**a**) RD 769 cell line, (**b**) MDA-MB-231 cell line, (**c**) A549 cell line, (**d**) CCD-19Lu cell line without laser, and (**e**) 5 minute 2 W cm^{-2} 808 nm laser irradiation treatment for different cells without nanoparticle. Scale bar: 275 μm.

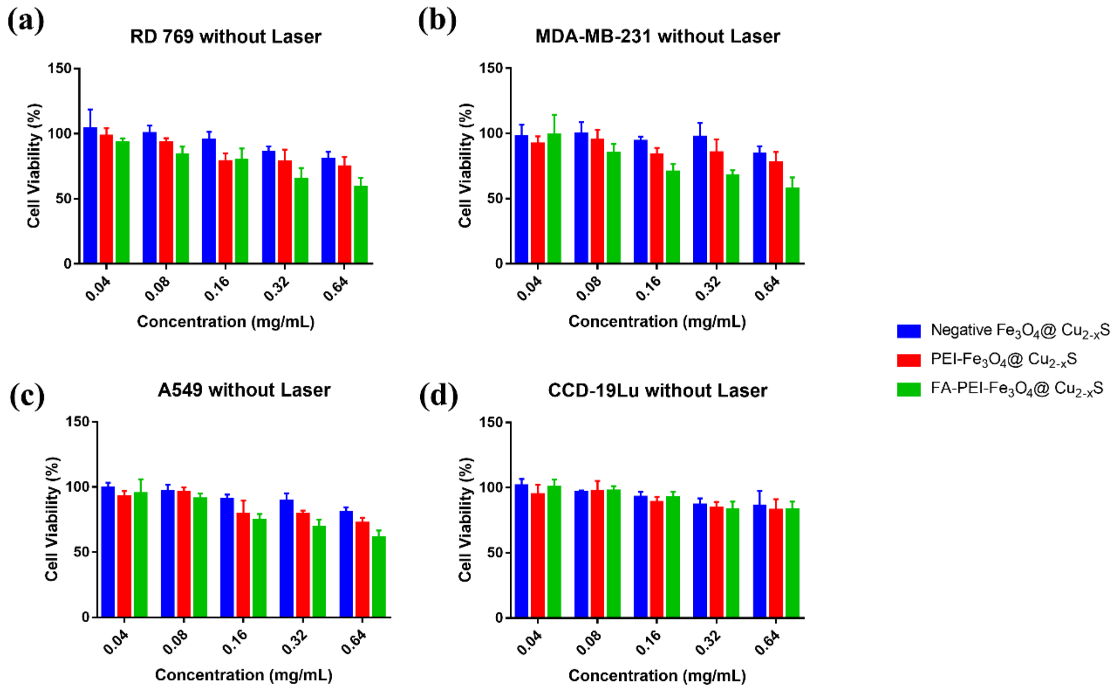

Figure S6. Cell viability vs. concentration of different nanoparticles for cancer cells (**a**) RD 769, (**b**) MDA-MB-231, (**c**) A549 and (**d**) non-malignant CCD-19Lu cells without laser treatment.
